# Recovery of the Foot Loading Patterns of Children with Excess Weight after Losing Weight: A 3-Year Longitudinal Study

**DOI:** 10.3390/children9050595

**Published:** 2022-04-22

**Authors:** Ruoyi Li, Xiaohong Sun, Shiyang Yan, Yihong Zhao, Jitka Badurova, Luming Yang, Haojun Fan

**Affiliations:** 1 National Engineering Research Center of Clean Technology in Leather Industry, Sichuan University, Chengdu 610065, China; 2017323080014@stu.scu.edu.cn (R.L.); shiyangy@kth.se (S.Y.); zhaoyihong@stu.scu.edu.cn (Y.Z.); 2 Key Laboratory of Leather Chemistry and Engineering, Sichuan University, Chengdu 610065, China; fanhaojun@scu.edu.cn; 3 Yantai Affiliated Hospital of Binzhou Medical University, Yantai 256699, China; hezhenge@stu.scu.edu.cn; 4 Faculty of Technology, Tomas Bata University, 76001 Zlin, Czech Republic; badurova@utb.cz

**Keywords:** weight loss, overweight, obese, foot loading patterns, load transfer, longitudinal study

## Abstract

It is suggested that children with excess weight should lose weight to reduce plantar pressure and the risk of related injuries. However, whether the foot loading patterns of these children could return to normal after weight loss is unclear. A total of 147 children participated in this longitudinal study; 51 were selected for analysis—13 children who were overweight and 1 child with obesity reduced their weight to normal levels and 37 children maintained normal weights (control group). The plantar pressure parameters, including peak pressure, maximum force, and force-time integral were recorded using a Footscan plate system. Comparisons of plantar parameters and load transferences revealed that weight loss could effectively decrease the differences in foot loading distributions between the weight-reduced and normal-weight groups. After losing weight, the foot loading patterns of the children who were overweight recovered to the level of normal-weight children, and that of the child with obesity failed to reach the normal level. Losing weight is suggested for children who are overweight/obese to recover their foot loading patterns, to avoid further adverse influences on the foot/functioning caused by excessive weight-bearing. Further research exploring the findings of a cohort of children with obesity—who reduce their weight to normal levels—is warranted.

## 1. Introduction

Childhood obesity is related to higher plantar pressure and lower motor skills [1,2,3,4], which leads to an increased risk of lower extremity exercise-related pain and overuse injuries in such children, compared to children with normal weight [5,6]. Many researchers and clinicians suggest children with excess weight lose weight via lifestyle interventions or bariatric surgery [7,8], which could mitigate the negative impact of excess body mass on the feet and walking gait [9,10,11].

The foot arches and musculoskeletal systems in children are immature and are subject to developmental processes [12,13,14,15]. As the foot is not fully developed, the foot structure and foot functioning are susceptible to childhood obesity, which causes a flattened midfoot region and higher dynamic plantar pressures in children who are overweight and obese [16,17,18]. Losing weight has been found to be an effective method to reduce plantar pressure [11,19]. A previous longitudinal study indicated that weight reduction in children with obesity could effectively decrease both static and dynamic plantar pressures [11]. However, this study only compared follow-up data with baseline data. Given the short follow-up time of a few months, the time to reduce to normal weight was not sufficient for children with obesity. Hence, it is unclear whether the foot loading patterns of children with obesity can return to the normal patterns after weight loss. One may wonder if the different foot loading patterns would persist after the weight returns to normal. Therefore, a long-term follow-up study was conducted, which allowed children who were overweight and obese to achieve normal weight. The foot loading patterns between the two groups of children and normal-weight children were compared to investigate whether they could recover to the levels of normal-weight children. Additionally, a load transfer analysis was performed to gain insight into how the load transferred on feet as the overweight/obese children lost weight; the load transfer analysis was used in some previous research [20,21,22] and it was a useful tool, in the present study, to gain insight into how the load is redistributed with weight reduction.

Therefore, this study aimed to identify how foot loading redistributes with weight loss in children who are overweight and obese and whether these foot loading patterns can recover to the levels in normal-weight children over the three years.

## 2. Methods

### 2.1. Participants

In total, 147 children aged 7–9 years (82 boys and 65 girls) participated in the original study; they were recruited from a random local primary school in Yantai, China. A follow-up study was conducted after 3 years. All participants were screened via a physical examination and interview for injuries, disease, foot deformity, or abnormal gait (for example, hallux valgus, and toe-out gaits). Participants with lower extremity injuries, previous foot surgeries, or biomechanical abnormalities that affected their gait were excluded. The guardians of all children signed informed consent forms prior to the procedure. This study was conducted in accordance with the Declaration of Helsinki. Ethical approval was obtained from the Ethics Committee of Sichuan University (ID number: K2020044). 

Body mass index (BMI) was calculated as weight/height^2^ (kg·m^−2^). The weight and height of all children were measured at the baseline and follow-up by the experimenter using an electronic scale and stadiometer, respectively. Participants were divided into three categories: obese, overweight, and normal weight, according to the BMI reference norm, which was established by the Group of China Obesity Task Force [23]. Table 1 shows the BMI reference norm at 7–12 years, which was used to classify the BMI of children in the present study. Except for the regular exercises in physical education class (approximately 2 h/week), no intervention was performed during the 3 years. At the 3-year follow-up, children with excess weight who had lost weight to achieve normal weight and those who had maintained normal weight were selected. Finally, this study included 51 children: (a) 13 children with excess weight who had normal weight after weight loss (OV-N); (b) 37 children with normal weight who maintained their weight (N-N); and (c) one child with obesity who had normal weight after weight loss (O-N). It is worth noting that, due to the long-time span of this study, the weight and height of the participants changed significantly; therefore, whether the children lost weight effectively was based on the BMI categories. Weight loss in this study should be considered as the reduction of obesity degree rather than the reduction of body weight, which can be identified as ‘relative weight loss’. 

### 2.2. Equipment and Procedure

All recruited children participated in the measurements at baseline and follow-up. Anthropometric data were recorded, including sex, age, height, and weight. Plantar pressures were recorded using a one-meter Footscan plate system (RSscan International, Olen, Belgium) with a sampling frequency of 250 Hz. Data on pressure parameters, including peak pressure, maximum force, and force-time integral (FTI) were collected. The plate was set on a firm and level surface, with two 5-m rubber walkways connected to both ends. A two-step initial protocol was used to perform the experimental procedures [24]. After acclimatization, children were asked to walk naturally and barefooted at their preferred speed across the walkway. The plate system collected the plantar parameter data as a trial as the participants walked across the platform. A trial was considered successful if the participant walked naturally through the walkway at a self-preferred speed and left two whole steps of both feet on the plate system. At least three valid trials were collected for each child.

### 2.3. Data Processing

The plantar region of the foot was divided into 10 anatomical regions [25,26]: big toe (BT), second-fifth toes (T2-5), first metatarsal (M1), second metatarsal (M2), third metatarsal (M3), fourth metatarsal (M4), fifth metatarsal (M5), midfoot (MF), medial heel (HM), and lateral heel (HL). The average of the plantar pressure parameters of three valid trials was calculated for each child.

Peak pressure represents the aggregate of all of the measured pressures from different sensors in specific parts of the foot [27], which is important when investigating foot injury mechanisms [5,28].

Absolute body weight has been found to correlate with plantar forces [29]. To eliminate the influence of body weight on plantar pressures in different groups, the maximum force was normalized to the product of the body mass time gravity of individuals. The normalized maximum force was used to assess whether the foot loading patterns of children who were overweight and obese could return to the normal patterns after relative weight loss. The normalized maximum force was calculated as bellow (*g* refers to gravity):

(1) $$\mathrm{Norm}\;\mathrm{MF}\;\left(\%\right)=\frac{\mathrm{MF}}{\mathrm{Body}\;\mathrm{weight}\times g}\times 100\%,$$

The Arch index (AI) is calculated as a tool to compare the arch height between the groups. The AI is calculated as the ratio between the contact area in MF and the contact area of the total foot without the toes [30].

(2) $$\mathrm{AI}=\frac{\mathrm{CA}\left(\mathrm{midfoot}\right)}{\mathrm{CA}\left(\mathrm{total}\;\mathrm{plantar}\;\mathrm{regions}\;\mathrm{without}\;\mathrm{the}\;\mathrm{toes}\right)},$$

FTI is the total load of a certain area of the foot and represents the duration of contact, so the overall load of these regions can be determined [22]. Hence, the FTI of each region was calculated to assess the inter-regional load transfer [22]. To eliminate the influence of various body masses between groups, the FTI was normalized to the relative FTI (FTIrel) as follows:

(3) $$\mathrm{FTIrel}\;\left(\%\right)=\frac{\mathrm{FTI}\left(\mathrm{foot}\;\mathrm{region}\right)}{\sum \mathrm{FTI}\left(\mathrm{All}\;\mathrm{foot}\;\mathrm{regions}\right)}\times 100\%,$$

### 2.4. Load Transfer

Load transfer with relative weight loss in children who were overweight and obese was assessed by a load transfer analysis method, which was used in many previous studies [22,31,32]. The transfer values over the three years were calculated as FTIrel values at the baseline minus the FTIrel values at the follow-up. The positive values mean that the foot load transferred out of this foot region, and the negative values mean that the load shifted to this region from other regions. Four parts of the foot were divided according to the anatomical segments: level 1: toes (BT and T2-5), level 2: metatarsal (M1, M2, M3, M4, and M5), level 3: midfoot (MF), and level 4: heel (HM and HL).

### 2.5. Statistical Analysis

SPSS 21.0 (IBM SPSS, IBM Corp., Armonk, NY, USA) was used to perform statistical analyses. An independent t-test showed that no significant difference was found in foot loading between the left and right feet (*p* > 0.05). As most participants were right foot dominant, the foot loading of the right foot was used for analysis in this study. The Kolmogorov–Smirnov test and Q–Q normality plots were applied to assess data normality (*p* > 0.05). The differences in the values of anthropometric variables and foot loading parameters at baseline and follow-up were evaluated using an independent *t*-test for both OV-N and N-N. To explore whether anthropometric variables and the foot loading patterns of OV-N could return to the level of normal-weight children, statistical differences between OV-N and N-N were identified using an independent t-test at baseline and follow-up. The one-sample t-test was performed to compare the differences between O-N and N-N because of only one case of O-N. The results of the partial correlation analysis revealed no significant correlations between age and FTIrel; therefore, the effect of age on load transfer was negligible in the present study. The *p* value < 0.05 was regarded as significant for all statistical analyses. Meanwhile, the effect size (ES) was calculated using Cohen’s d for the t-test for significant differences. The following interpretations of ES were used: trivial (0–0.2), small (0.2–0.6), moderate (0.6–1.2), and large (>1.2).

## 3. Results

### 3.1. Participant Characteristics

A total of 37 N-N children, 13 OV-N children, and 1 O-N child were analyzed in the present study. The participant characteristics are shown in Table 2. The height and weight of all children increased significantly with the overall development over 3 years. Although the weights of the children who went from overweight and obese levels to normal-weight levels were higher than that of normal-weight children, their BMIs had reached the normal levels. No significant difference was found in the arch index between OV-N and N-N before and after relative weight loss. However, the arch index of the O-N child was significantly greater than those of N-N children both before and after relative weight loss.

### 3.2. Plantar Pressure

The peak pressure and normalized maximum force in all children are presented in Table 3. Although no significant difference was found in peak pressures between OV-N and N-N at baseline, peak pressures were significantly greater in the M3 and M4 regions in OV-N than in N-N at follow-up. The normalized maximum forces significantly decreased in the T1 and T2-5 regions in OV-N than in N-N at baseline; however, this reversed to the level in N-N at the follow-up. At baseline, peak pressures were greater in all plantar regions in the O-N child compared with N-N children; however, peak pressures were greater only in the T1, M1, M2, M3, and HM regions in the O-N child compared with N-N children after relative weight loss. At baseline, the normalized maximum forces were significantly greater in the T2-5, M3, M4, M5, MF, and HM regions in OV-N compared with N-N children. After losing weight, the normalized maximum forces were significantly greater in the M2, M3, and HM regions in OV-N children compared with N-N children, whereas the normalized maximum force was significantly lower in the M5 region in OV-N children compared with N-N children. 

### 3.3. Load Transfer

**Figure 1:**
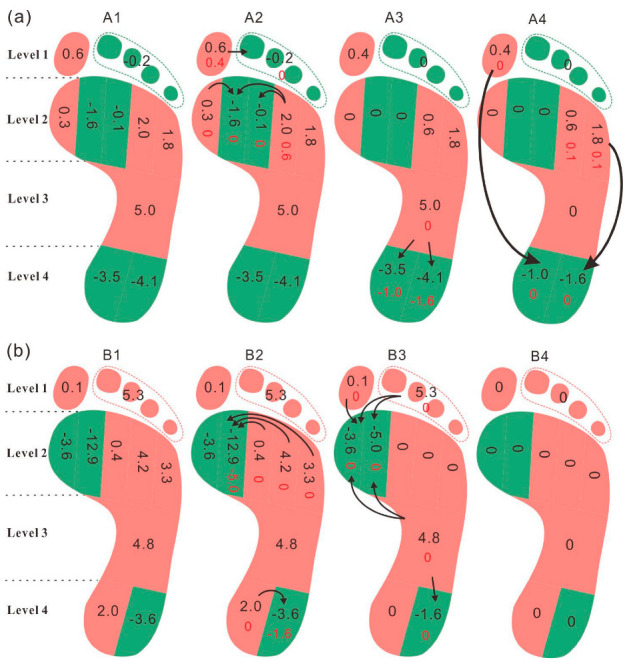
(**a**) Load transfer with relative weight loss in children who were overweight; (**b**) load transfer with relative weight loss in children who were obese. Figure 1 legend: A1 and B1 show the transfer values of FTIrel in each foot region. A2 and B2 display the load transfers within each foot level. A3 and B3 show the load transfers between the adjacent foot levels. A4 and B4 display the load transfers across foot levels. The arrows represent the transfer of foot loading, from the foot regions in red (positive value) to the foot regions in green (negative value).

The FTIrel of each foot region and the transfer values are listed in Table 4. Changes in load transfer with relative weight loss in children who were overweight and obese are shown in Figure 1. At baseline, the FTIrel was significantly greater in the M4 region and significantly lower in the T2-5 region in OV-N compared with N-N. After the relative weight loss, the foot load mainly transferred to M2, M3, HM, and HL regions in the OV-N children. Analysis of foot loading revealed that the load was concentrated in the M2 and M3 regions and transferred from the midfoot and metatarsal regions to the heel. After relative weight loss, the significant differences in the FTIrel in the T2-5 and M4 regions between OV-N and N-N disappeared; however, the FTIrel remained greater in the M3 region in OV-N than in N-N after loss. 

The FTIrel was greater in the T2-5, M3, and M4 regions and lower in the BT and HL regions at baseline in the O-N child compared with N-N children. After the relative weight loss, a lateral-to-medial load transfer (from the M3, M4, and M5 to the M1 and M2) and a transfer from MF to metatarsal and the heel of foot loadings were observed in the O-N child. The FTIrel was greater in the M2 and M3 regions and lower in the T2-5 and M5 regions in the O-N child compared with N-N children at follow-up. Notably, foot loading transferred out of the MF regions after relative weight loss in both OV-N and O-N.

## 4. Discussion

In this longitudinal study, changes in foot loading patterns with relative weight loss were assessed and the foot loading patterns of children who went from overweight and obese levels to normal-weight levels were compared with those who maintained a normal weight. The result revealed that peak pressure in the heel regions increased significantly with the gain of age in children who were overweight, as well as foot loading concentrated in the heel regions with the load transfer. The OV-N children displayed a different developmental pattern with N-N children, whose heel regions showed a disproportionate increase of plantar pressure. No significant change was noted in the normalized maximum force. After relative weight loss, no significant difference was found between the normalized maximum force in children with excess weight and that in children with normal weight. The difference in the foot loading pattern between the child with obesity and children with normal-weight levels reduced after relative weight loss; however, the foot loading pattern of the child with obesity failed to reach the level of the normal-weight children. 

Compared to the normal-weight children, no significant difference was found in the baseline peak pressure data of children who were overweight. However, surprisingly, the follow-up peak pressures were significantly greater in the third metatarsal and fourth metatarsal regions in those who reduced weight. At the 3-year follow-up, the body mass of N-N and OV-N children increased to 9.6 and 13.8 kg, respectively. Although no significant difference was found in the BMI between the two groups at follow-up, the body weight of OV-N was significantly higher than that of N-N. Increased body weight has been shown to elevate peak pressure [33]. Hence, the peak pressures in the main loading regions were greater in children who were overweight compared to those with normal weight, even after losing weight to the normal level. Generally, plantar pressures reduce with weight loss [11,19]. However, due to the long-time span in this study, the weight and height of the participants have changed significantly; therefore, whether children lose weight effectively is based on the BMI categories. Weight loss in this study should be considered as the reduction of obesity degree rather than the reduction of body weight, which can be identified as ‘relative weight loss’. Actually, the weight of the children in all groups increased over the three years (Table 2). It was noticed that peak pressure in the heel regions increased significantly with the gain of age in OV-N, accompanied by the load transferred from the toes, metatarsal, and midfoot to the heel regions. Different development patterns of plantar pressure were displayed between OV-N and N-N with the gain of age. Although the peak pressure values and the development patterns of peak pressure were significantly different between OV-N and N-N, no significant difference was found in the normalized maximum force between children with excess and normal weights after normalizing the plantar forces to body mass. This suggests that the foot loading patterns of children who were overweight recovered to the level of children with normal weight in all plantar regions after losing weight. Additionally, the normalization of maximum forces successfully eliminated the influence of body mass on foot loadings in children who were overweight.

The obese case observed in this study displayed higher peak pressures in all plantar regions and a greater arch index. The results were in accordance with the foot characteristics of children with obesity in previous studies [5,28,34,35]. The higher pressure was related to higher risks of foot discomfort, foot deformity, foot pain, and other injuries [5,28], and the greater arch index was associated with a lower medial longitudinal arch [34,35]. After the relative weight loss, the foot loading patterns and the arch index of the child with obesity remained different from those of normal-weight children, which may suggest that the foot structure and foot function of the child with obesity may have changed. Although the foot loading patterns and arch index of the child with obesity failed to reach the level of normal-weight children in this study, changes indicating adaptation to the decreased body weight and toward the normal level were noted, such as the transference of foot loading from the midfoot region to other regions and elevation of the foot arch. To figure out why the child with obesity could not recover to the normal level after losing weight, the authors propose two possible reasons: first, the time was not sufficient for the human body to adjust the foot loading patterns to the reduced weight; second, repetitive and long-term overload on the feet resulted in rigid deformation of the foot structure, causing the foot loading patterns to fail to recover to normal, even after relative weight loss. Further research investigating the findings of a cohort of children with obesity—who reduce their weight to normal levels—is warranted.

The mechanism of redistribution of the plantar load with relative weight loss was investigated using the load transfer analysis method in the present study. Weight loss resulted in the redistribution of plantar loading between the plantar regions in children who were overweight and obese. It is noteworthy that load transference accompanied by relative weight loss could effectively relieve plantar loadings. As shown in the results, the foot loading in the fourth metatarsal region was significantly greater in OV-N children than that in N-N children. Weight reduction resulted in the transfer of the load borne by the fourth metatarsal region to the second and third metatarsal regions, and further to the heel in children who were overweight. The difference in the FTIrel in the fourth metatarsal between children with excess weight and normal weight decreased from 2.6% to 2.1% after load transfer. Meanwhile, foot loading transferred from the big toe to the second-fifth toes where the FTIrel was significantly lower than that in normal-weight children. The difference in the FTIrel in the second-fifth toes region between children with excess weight and those with normal weight decreased from 1.2% to 0.1%. After the load transference, the differences between children with excess weight and those with normal weight tended to decrease. When it came to the child with obesity, greater FTIrel was found in the T2-5, M3, and M4. The foot loads transferred from T2-5 to the BT and from the M3 and M4 to the M2, respectively. In summary, in the process of the relative weight loss, the differences between children who were overweight/obese and children with normal weight decreased with load transference, which eliminated the differences in foot loading distributions compared with normal-weight children. This might be a load recovery mechanism in the plantar after relative weight loss in children who are overweight and obese, which reverses the foot loading distribution to the normal pattern. Additionally, the transfers of foot loading from the midfoot region to the metatarsal and heel in the child with obesity might explain the former finding that the normalized maximum force in the midfoot region in the child who is obese decreased to the normal level with the relative weight loss. After the transfer of foot loading, the normalized maximum force in the midfoot region in the child with obesity decreased from 29.4% to 16.4%. Although the foot loading pattern of the child with obesity remained different from that of children with normal weight, it was closer to the normal level upon assessing the redistribution of plantar loading.

Our study has three strengths. First, a 3-year longitudinal investigation was conducted in this study, which allowed sufficient time for children who were overweight and obese to lose weight to the normal level. Second, the comparison of foot loading between children who went from being overweight/obese to normal weight and children with normal weight allowed us to identify whether the foot loading distribution in children who are overweight/obese could return to the normal pattern after relative weight loss. Third, a load transfer analysis method was applied to gain insight into the mechanism of load transference after the relative weight loss. However, this study has two limitations. First, a study with a larger sample size should be conducted to reinforce the results of this study, especially those in the case of obesity. Only one obese child who lost weight to a normal level was observed in this study; hence, it is still unclear whether the plantar pressure patterns of children with obesity can reach the level in those with normal weight after weight loss. Repeated follow-ups are recommended to investigate whether the foot loading pattern and foot structure change with long-time adaptation to weight-bearing. Second, this was an observational study, rather than an interventional study; therefore, the result of the present study cannot completely replace the effect of weight loss intervention.

## 5. Conclusions

In the present study, the foot loading patterns of children who were overweight—but reduced their weight to normal levels—recovered to the levels of children with normal weight. The foot loading patterns in the obese case showed higher foot pressures after the relative weight loss. Comparisons of plantar parameters and load transfers revealed that relative weight loss could effectively decrease the differences in foot loading distributions between the weight-reduced and normal-weight groups. The foot loading patterns reversed to the levels of normal-weight children, with weight loss, in the present study; therefore, the authors suggest losing weight to recover foot loading patterns, to prevent further adverse effects on the foot/functioning caused by excessive weight-bearing. Further research (with a larger cohort) exploring the effects of weight loss interventions on foot loading patterns—in children who reduce their weight to normal levels—is warranted. 

## Figures and Tables

**Table 1:** China BMI reference norm (7–12 years).

Age	Male (kg·m^−2^)		Female (kg·m^−2^)	
	Overweight	Obese	Overweight	Obese
7	17.4	19.2	17.2	18.9
8	18.1	20.3	18.1	19.9
9	18.9	21.4	19.0	21.0
10	19.6	22.5	20.0	22.1
11	20.3	23.6	21.1	23.3
12	21.0	24.7	21.9	24.5

**Table 2:** Participant characteristics (mean ± SD).

	Group	Baseline	Follow-Up	Mean Difference (95% CI)	*p*
Number	N-N	37	37	-	-
	OV-N	13	13	-	-
	O-N (one case)	1	1	-	-
Age (y)	N-N	8.1 ± 0.8	11.1 ± 0.8	3	-
	OV-N	8.0 ± 0.8	11.0 ± 0.8	3	-
	O-N (one case)	8.0	11.0	3	-
Height (cm)	N-N	130.5 ± 6.9	149.5 ± 7.8	19.0 (17.4 to 20.7)	0.000
	OV-N	131.2 ± 5.2	153.5 ± 7.9	22.3 (19.7 to 24.9)	0.000
	O-N (one case)	136.1	150.0	13.9	-
Weight (kg)	N-N	28.7 ± 6.3	38.3 ± 7.5	9.6 (7.5 to 11.7)	0.000
	OV-N	**32.4 ± 3.3**	**46.2 ± 5.4**	13.8 (11.9 to 15.6)	0.000
	O-N (one case)	**37.1**	**44.8**	7.7	-
BMI (kg·m^−2^)	N-N	16.7 ± 2.0	17.0 ± 2.2	0.3 (−0.3 to 1.0)	0.499
	OV-N	18. 8 ± 0.7	19.5 ± 0.9	0.8 (0.3 to 1.2)	0.028
	O-N (one case)	20.0	19.9	−0.1	-
AI	N-N	0.30 ± 0.03	0.28 ± 0.04	−0.02 (−0.03 to 0.00)	0.066
	OV-N	0.30 ± 0.03	0.29 ± 0.04	−0.01 (−0.04 to 0.02)	0.395
	O-N (one case)	**0.34**	**0.31**	−0.03	-

*p* values represent the differences between the baseline and the follow-up at each group. The **bold** represents significant differences between OV-N/ O-N and N-N at baseline or follow-up.

**Table 3:** Peak pressure (N/cm2) and normalized maximum force (%).

Regions	N-N			OV-N				O-N (One Case)		
	Baseline	Follow-Up	^a^*p*, ES	Baseline	Follow-up	^a^*p*, ES	^b^*p*, ES	Baseline	Follow-Up	^b^ *p*
Peak pressure (N/cm^2^)										
BT	4.3 ± 1.5	5.3 ± 3.0	0.075	3.9 ± 1.2	4.7 ± 2.8	0.344	0.571	**7.7**	7.1 ^b^	0.001
T2-5	1.2 ± 0.5	1.0 ± 0.8	0.269	0.9 ± 0.3	1.0 ± 0.6	0.809	0.977	**3.5**	0.5 ^b^	0.000
M1	3.8 ± 1.2	5.4 ± 2.7 ^a^	0.001, 0.77	3.6 ± 1.2	5.0 ± 3.1	0.149	0.653	**7.0**	6.4 ^b^	0.049
M2	7.6 ± 2.1	9.5 ± 3.7 ^a^	0.007, 0.63	7.4 ± 2.5	10.6 ± 5.7	0.080	0.454	**11.5**	17.6 ^b^	0.000
M3	8.3 ± 2.3	8.4 ± 4.1	0.901	9.6 ± 3.1	12.1 ± 5.8 ^b^	0.197	0.016, 0.74	**14.8**	13.8 ^b^	0.000
M4	6.9 ± 2.7	6.5 ± 3.7	0.592	8.0 ± 2.5	10.1 ± 6.6 ^b^	0.292	0.019, 0.67	**12.3**	7.1	0.338
M5	3.0 ± 1.4	3.1 ± 2.3	0.701	3.4 ± 1.2	4.5 ± 4.2	0.403	0.163	**4.1**	2.2 ^b^	0.014
MF	2.0 ± 0.9	1.8 ± 1.3	0.529	2.3 ± 0.7	2.3 ± 1.4	0.888	0.363	**3.2**	1.9	0.966
HM	7.3 ± 1.8	8.8 ± 4.6	0.071	7.4 ± 1.9	11.2 ± 4.7 ^a^	0.013, 1.06	0.116	**11.8**	11.1 ^b^	0.005
HL	6.6 ± 1.5	8.3 ± 4.3 ^a^	0.024, 0.53	6.6 ± 1.4	10.6 ± 6.0 ^a^	0.033, 0.92	0.161	**7.9**	9.6	0.099
Normalized maximum force (%)										
BT	20.6 ± 8.1	19.7 ± 10.5	0.687	**16.2 ± 5.0**	14.2 ± 8.5	0.469	0.096	18.9	17.9	0.311
T2-5	7.5 ± 4.0	4.9 ± 3.7 ^a^	0.005, 0.67	**4.9 ± 1.6**	4.0 ± 2.5	0.302	0.430	**19.1**	1.7 ^b^	0.000
M1	19.5 ± 7.4	24.0 ± 14.4	0.097	16.7 ± 6.9	17.8 ± 12.1	0.776	0.174	19.2	26.8	0.241
M2	20.0 ± 5.8	26.3 ± 9.1 ^a^	0.001, 0.83	19.3 ± 6.5	23.7 ± 11.8	0.260	0.415	20.7	47.4 ^b^	0.000
M3	19.9 ± 4.2	18.0 ± 6.9	0.139	22.7 ± 6.5	23.1 ± 10.8	0.917	0.055	**26.3**	23.7 ^b^	0.000
M4	16.1 ± 5.3	12.6 ± 7.2 ^a^	0.018, 0.55	18.6 ± 5.7	17.4 ± 12.0	0.748	0.092	**21.7**	11.2	0.252
M5	8.6 ± 3.8	6.1 ± 4.5 ^a^	0.011, 0.60	9.4 ± 3.9	6.8 ± 4.9	0.147	0.642	**10.1**	3.3 ^b^	0.001
MF	22.2 ± 9.8	17.0 ± 12.8	0.057	24.2 ± 9.5	18.3 ± 15.1	0.247	0.771	**29.4**	16.4	0.771
HM	35.9 ± 7.2	34.6 ± 18.9	0.698	33.6 ± 10.5	40.3 ± 18.9	0.276	0.357	50.6 ^b^	41.3 ^b^	0.038
HL	28.2 ± 4.8	30.8 ± 15.8	0.336	26.2 ± 7.2	34.5 ± 23.4	0.234	0.537	27.6	32.8	0.456

^a^*p* represent the differences in foot loading parameters between the baseline and the follow-up at each group; ^b^ *p* represent the differences in follow-up foot loading parameters between OV-N/ O-N and N-N. The **bold** represents statistical differences in baseline plantar pressure parameters between OV-N/O-N and N-N.

**Table 4:** FTIrel (%) and the transfer values.

Regions	N-N		OV-N			O-N (One Case)		
	Baseline	Follow-Up	Baseline	Follow-Up	Transfer Value	Baseline	Follow-Up	Transfer Value
BT	9.5	8.8	7.2	6.6	0.6	7.6 ^a^	7.5	0.1
T2-5	3.0	1.9	1.8 ^a^	2.0	−0.2	5.8 ^a^	0.5 ^b^	5.3
M1	10.1	13.7	8.8	8.5	0.3	8.9	12.5	−3.6
M2	10.9	15.4	10.8	12.4	−1.6	10.8	23.7 ^b^	−12.9
M3	11.0	10.5	13.3	13.4 ^b^	−0.1	12.9 ^a^	12.5 ^b^	0.4
M4	9.0	7.5	11.6 ^a^	9.6	2.0	11.0 ^a^	6.8	4.2
M5	4.3	3.2	5.0	3.2	1.8	4.9	1.6 ^b^	3.3
MF	11.3	8.7	13.5	8.5	5.0	11.5	6.7	4.8
HM	17.5	16.2	16.1	19.6	−3.5	17.7	15.7	2.0
HL	13.5	14.0	12.0	16.1	−4.1	9.0 ^a^	12.6	−3.6

^a^*p* represents statistical differences in baseline FTIrel values between OV-N/O-N and N-N; ^b^ *p* represents statistical differences in follow-up FTIrel values between OV-N/O-N and N-N.

## Data Availability

The data presented in this study are available upon request from the corresponding author.
